# Adherence to additional medication for management of HIV-associated comorbidities among older children and adolescents taking antiretroviral therapy

**DOI:** 10.1371/journal.pone.0269229

**Published:** 2022-06-15

**Authors:** Andrea M. Rehman, Victoria Simms, Grace McHugh, Hilda Mujuru, Lucky G. Ngwira, Robina Semphere, Brewster Moyo, Tsitsi Bandason, Jon O. Odland, Rashida A. Ferrand

**Affiliations:** 1 MRC International Statistics and Epidemiology Group, London School of Hygiene & Tropical Medicine, London, United Kingdom; 2 Biomedical Research and Training Institute, Harare, Zimbabwe; 3 Department of Paediatrics, University of Zimbabwe, Harare, Zimbabwe; 4 Department of Clinical Sciences, Liverpool School of Tropical Medicine, Liverpool, United Kingdom; 5 Malawi-Liverpool-Wellcome Trust Clinical Research Programme, Blantyre, Malawi; 6 Department of Microbiology & HNTI, College of Medicine, Blantyre, Malawi; 7 Department of Public Health and Nursing, Norwegian University of Science & Technology, Trondheim, Norway; 8 School of Health Systems and Public Health, Faculty of Health Sciences, University of Pretoria, Pretoria, South Africa; 9 Department of Clinical Research, London School of Hygiene & Tropical Medicine, London, United Kingdom; PLOS, UNITED KINGDOM

## Abstract

**Background:**

Management of co-morbidities among persons living with HIV is an emerging priority, which may require additional medication over and above life-long antiretroviral therapy (ART). We explored factors associated with adherence to the trial drug among children and adolescents with perinatally acquired HIV taking antiretroviral therapy (ART) in the Bronchopulmonary Function in Response to Azithromycin Treatment for Chronic Lung Disease in HIV-Infected Children (BREATHE) trial.

**Methods:**

The BREATHE trial recruited 6–19 year olds with perinatally acquired HIV and co-morbid chronic lung disease as measured by FEV_1_. This two-site trial was individually randomised (1:1), double-blind and placebo-controlled. Participants received a once-weekly weight-based dose of 1–5 tablets of azithromycin (AZM: 250mg) or placebo, taken orally. We used pharmacy dispensing records and count of returned pills to measure adherence to study medication. Logistic regression was used to explore factors associated with adherence coverage. Poisson regression with Lexis expansion for time was used to explore whether adherence modified the effect of azithromycin on the incidence of acute respiratory exacerbation, a secondary outcome of the trial. Trial registration: ClinicalTrials.gov NCT02426112.

**Results:**

The 347 participants (median age 15.3, 51% male) consumed 14,622 doses of study medication over 16,220 person-weeks under study. Adherence was higher for those randomised to AZM (73.4%) than placebo (68.4%) and declined over the 48 weeks of the study (Score test for trend <0.02). Those with unsuppressed HIV viral load at baseline had 2.08 (95% CI: 1.19, 3.63) times the odds of non-adherence than those with viral suppression. Differences were also observed between trial sites.

**Conclusion:**

The majority of children and adolescents tolerated the addition of a once-weekly dose of medication to their pill burden. Barriers in adhering to treatment for co-morbid conditions are likely common to barriers in adhering to ART. Control of co-morbidities will therefore present additional challenges in HIV care.

## Introduction

The scale-up of paediatric anti-retroviral therapy (ART) programmes in sub-Saharan Africa has resulted in increasing numbers of children accessing treatment and also surviving to reach adolescence and adulthood [1]. However, there is increasing recognition that children growing up with HIV are at high risk of a range of multisystem co-morbidities despite ART [2]. Co-morbidities such as chronic lung disease, chronic kidney disease and cardiac disease may be a consequence of HIV infection itself or its treatment.

Multimorbidity, commonly defined as the co-existence of two or more chronic health conditions [3] increases the complexity of therapeutic management. In the context of HIV infection which requires life-long ART, management of associated comorbidities will require polypharmacy. or the use of multiple medicines may result in adverse effects due to multiple factors including drug interactions, side-effects and increased pill burden [4]. The latter may be particularly difficult for older children and adolescents who have poor adherence to drug regimens for many chronic illnesses [5]. Multiple studies have demonstrated that this age-group has worse HIV treatment outcomes compared to other age-groups, partly due to poorer adherence to ART [6–8]. Adolescence is characterised by physiological and psychological development, increased desire for independence from parents, and increased risk-taking [9]. Adolescents also lack financial autonomy, are prone to peer pressure, and have limited problem-solving skills, which affect adherence to medication [9]. Further, in resource-limited settings, external factors including poverty, food scarcity, and HIV-related stigma influence ART adherence and HIV outcomes [10].

Adherence to medication for co-morbidities has not been previously examined among children and adolescents taking ART. We evaluated adherence to medication among 6–19 year-olds established on ART within an individually randomised double-blinded, placebo-controlled trial of weekly oral azithromycin given over a 48-week period for chronic lung disease. We investigated factors associated with adherence to trial medication and explore how adherence coverage, both overall and by dispensing period, affected the incidence of acute respiratory exacerbations, a clinical outcome of the trial which differed by trial arm.

## Materials and methods

### Study design and participants

The Bronchopulmonary Function in Response to Azithromycin Treatment for Chronic Lung Disease in HIV-infected Children (BREATHE) trial (registered at ClinicalTrials.govNCT02426112) showed no impact of azithromycin on lung function (Forced expiratory volume in 1 second—FEV_1_) but reduced incidence of acute respiratory exacerbations (AREs) among those receiving azithromycin [11]. Participants were recruited from outpatient HIV clinics at public-sector hospitals in Harare, Zimbabwe and Blantyre, Malawi and randomised with 1:1 allocation ratio stratified by site, to either azithromycin or placebo, with weight-based dosing [12]. In addition to study medication, the majority of participants were taking first-line ART, an NNRTI-based regimen, which is usually given as a fixed-dose combination requiring one pill per day. Cotrimoxazole prophylaxis was also common, and given either three times (2 pills each time) a week or a once daily dose (1 pill daily).

Perinatally HIV-infected 6–19 year olds were eligible for inclusion if they had been taking ART for at least six months and had chronic lung disease (defined as an FEV_1_ z-score <-1 with lack of reversibility (< 12% improvement with salbutamol 200μg inhaled using a spacer)), had a stable home address, guardian’s consent and age-appropriate participant assent (<18 year olds) or participant consent (18–19 year olds) and knowledge of their HIV status (for those aged 12 or over). Exclusion criteria included active lung infection (including tuberculosis (TB)), pregnancy, a prolonged QTc interval, creatinine clearance <30mls/minute, alanine amino transferase >2 times the upper limit of normal and presence of a fatal condition.

Ethics approval was obtained from the Malawi College of Medicine, the Medical Research Council of Zimbabwe, the Biomedical Research and Training Institute, Zimbabwe, the London School of Hygiene and Tropical Medicine, UK, the University of Cape Town, South Africa, and Medical and Health Research, Norway. Written informed consent was sought from the guardian, and age-appropriate written assent was sought from the participant (for those aged <18 years); those aged 18 years and older consented independently.

Participants, health care providers, dispensing pharmacists and outcome assessors were blinded to trial allocation. The main trial findings were calculated according to a pre-specified statistical analysis plan which included a definition of adherence determined prior to unmasking. At 48 weeks, there was no difference in mean FEV_1_ (primary outcome) between arms, but there was a 50% reduction in the risk of AREs [11].

### Measurement of adherence

Study medication was received in bottles of 60 tablets which were counted out by hospital pharmacists to provide the correct dose based on weight. Pharmacists dispensed oral azithromycin or placebo 250mg tablets, labelled by the pharmaceutical company as trial arm 1 or trial arm 2, into locally sourced containers of identical taste and appearance according to weight band (10–19.9kg: 1 tablet per week; 20–29.9kg: 2 tablets per week; 30–39.9kg:3 tablets per week; ≥40kg:5 tablets per week) at baseline, and 2-week, 12-week, 24-week and 36-week visits, or unscheduled visit (because of holidays or drug expiry). Weight was checked at each dispensing occasion and cross-checked by the pharmacist; changes in weight which necessitated a change in dose were communicated to participants. An extra two-week buffer dose was provided at each visit in case of delays to attending scheduled study visits. A household member was identified to directly observe consumption of study medication, and participants were provided with a diary to record their consumption which was retained by study personnel at the 48-week visit. An independent statistician provided monthly reports to the trial coordinator to identify potential irregularities in dispensing, such as incorrect treatment arm dispensing. If an error was found, participants were recalled to correct the error, dispensing procedures were reviewed and refresher training provided to staff. Participants were asked to return unused tablets, which should primarily be the buffer doses, at the next study visit.

For the purposes of defining an adherent participant we split time on study into four 12-week periods, 1) baseline-12 weeks, 13 doses, 2) 13–24 weeks, 12 doses 3) 25–36 weeks 12 doses, and 4) 37–48 weeks, 12 doses. We calculated the number of doses missed for each period based on the difference between the number of pills dispensed and the number of pills returned. If a participant did not return their buffer doses this could result in apparent over-consumption. For our primary outcome which was measured at 48 weeks after commencing study medication, we defined an adherent participant as not missing more than a mean of two doses over each of the four time periods, providing a measure of “good” adherence [11]. Periods where the participant was instructed to stop taking the study drug, per protocol, were counted as missed doses for the purpose of defining a sufficient dose of study medication to generate a biological response [13]. In time-to-event analysis where we compared those with good adherence against those without good adherence to explore the effect of adherence on trial outcomes, we used two definitions of good adherence. We compared the average good adherence described above with an alternative definition which was allowed to vary over time, with a participant coded as adherent for each of the four time periods separately if they did not miss more than two doses in that period.

At every study visit, participants were counselled about adhering to their study medication and to their ART. In addition, those found to be virally unsuppressed (HIV VL<1000 copies/ml) at baseline had the results fed back to the participant and to their provider and participants were referred for enhanced ART adherence counselling from their routine HIV care provider.

### Data management and statistical methods

Data were captured electronically using case report forms in OpenDataKit software running on Google Nexus^™^ tablets, (Google, Mountain View, CA, USA), and managed using a Microsoft Access database (Microsoft, Redmond, WA, USA) before conversion in Stata v16.1 software (StataCorp, Texas, USA) for analysis. Anonymised data was stored on secure SharePoint servers. A significance level of 0.05 was used for analysis.

HIV viral load was determined with XpertTM HIV-1 Viral Load (Cepheid, Sunnyvale, CA, USA), and suppression was defined as viral load <1000 copies/ml. Anthropometric z-scores were generated using the 1990 British reference equations [14], with underweight and stunted defined as a z-score for weight-for-age and height-for-age of <-2 respectively. Abnormal respiratory rates were determined by age-specific centiles developed in an Australian paediatric population [15].

Comparisons between good adherence and covariates were conducted using chi-square tests, and for ordered categorical covariates, such as time on study, a Score test for trend. We used logistic regression to investigate association of baseline factors were with adherence coverage. In adjusted models we included all covariates to explore the effects of confounding.

Poisson regression with random effects, to account for multiple acute respiratory exacerbation (ARE) events, and robust standard errors was used to calculate hazard ratios to explore whether the effect of AZM on the incidence of ARE among participants differed among adherent and non-adherent participants, by fitting an interaction term between trial arm and adherence. We did not use Cox regression models because the data violated the assumption of proportional hazards in this interaction model. A Lexis expansion, a method used to split follow-up time according to time-varying covariates, was used to account for effects of time on study, season of year and calendar time. Analysis time was split by follow-up time period (baseline to 12-weeks; 12–24 weeks; 24–36 weeks; and 36–48 weeks) to explore cumulative effects of treatment, season (rainy- November to April; dry—May to October) as AREs are known to increase in winter, and calendar time (2017–2018 and 2019–2020) to explore general trends over time. We additionally adjusted for site, age, sex and HIV viral load in alignment with our previously published results [11]. Participants were censored at date of death, date of withdrawal, date of last study visit (if lost to follow-up) or at 49 weeks after enrolment. Kaplan-Meier cumulative incidence curves were generated for graphical display.

## Results

**Fig 1:**
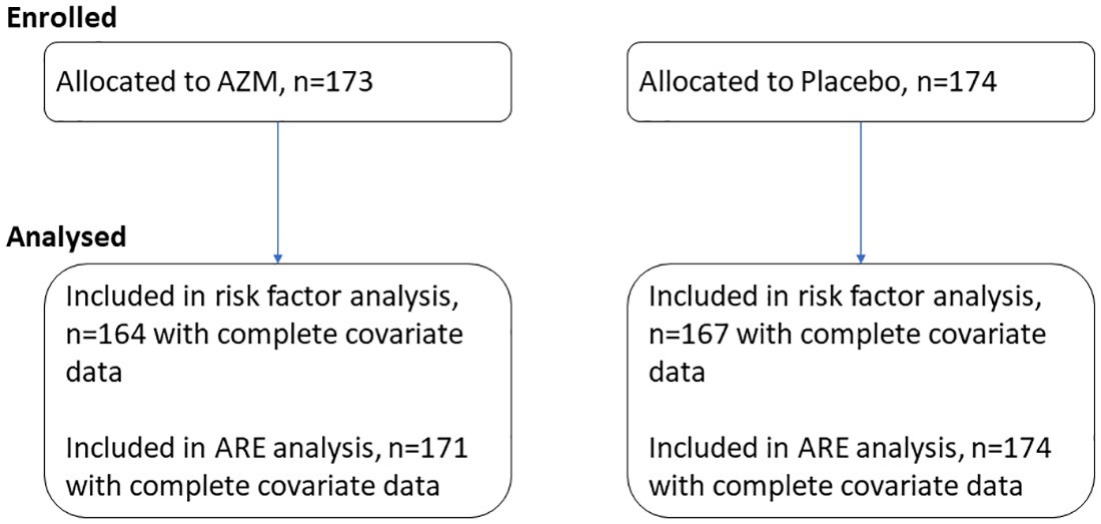
Participant flow.

All 347 enrolled participants, median age 15.3 (IQR 12.7, 17.7), 51% (n = 177) male, commenced study medication (Fig 1); participant characteristics have been reported previously [11]. Up to assessment for the primary outcome, participants attended 1,990 visits (median 6, range 1–6 per participant) over 16,220 person-weeks. Study medication was permanently stopped for eight participants per protocol after median 27 (IQR 10–38) weeks; three participants became pregnant, three experienced prolonged QTc interval, one commenced fluconazole, thus there was concern for drug-drug interaction and one experienced a possibly drug-related rash. Participants who withdrew (n = 15) or were lost to follow-up (n = 4) prior to 49 weeks, had unknown tablet consumption. There were three deaths, all in hospital, with known treatment stop dates.

Two participants accidentally received a dose of 4 tablets instead of 5 per week for 2 weeks early in the trial, one in each trial arm. A total of 35,520 placebo tablets (9,216 doses) and 33,570 azithromycin tablets (9,403 doses) were dispensed over the course of the trial (Table 1) with 7,197 placebo doses and 7,425 azithromycin doses presumed to be consumed. Pharmacists dispensed incorrect study medication for 103 person-weeks: six participants randomised to the placebo arm were incorrectly given azithromycin for a total of 73 person-weeks and three participants randomised to the azithromycin arm were incorrectly given placebo for a total of 30 person-weeks.

**Table 1:** Recorded dispensing and pill returns by study visit and trial arm.

Number of tablets (doses)	Trial arm	Enrolment	2w visit	12w visit	24w visit	36 w visit	Overall
**Dispensed**	Placebo	3038 (806)	7420 (1947)	8528 (2221)	8315 (2151)	8219 (2092)	35,520 (9216)
	AZM	2767 (790)	7059 (2014)	8070 (2289)	7946 (2189)	7728 (2121)	33,570 (9403)
**Presumed to be consumed**	Placebo	1732 (462)	5822 (1521)	6687 (1742)	6909 (1783)	6697 (1689)	27,847 (7197)
	AZM	1639 (466)	5444 (1563)	6475 (1869)	6443 (1770)	6401 (1756)	26,402 (7424)
**Returned**	Placebo	1173 (310)	1389 (364)	1385 (367)	1185 (301)	1132 (298)	6,264 (1661)
	AZM	1152 (325)	1343 (379)	1178 (324)	1323 (359)	1216 (335)	6212 (1722)
**Not accounted for**	Placebo	133 (34)	209 (62)	456 (112)	221 (67)	390 (105)	1409 (379)
	AZM	0 (0)	248 (71)	417 (95)	180 (60)	111 (30)	932 (255)
**Number of visits with apparent over-adherence (>115%)**	Placebo	-	18 (10.3%)	10 (5.8%)	12 (6.9%)	12 (6.9%)	52 (7.5%)
	AZM	-	15 (8.7%)	2 (1.2%)	7 (4.1%)	9 (5.2%)	33 (4.8%)

Apparent over-adherence is where the participant did not return the buffer doses, and was presumed to consume more than 15 doses in the first 12 weeks, or more than 14 doses in subsequent periods.

**Fig 2:**
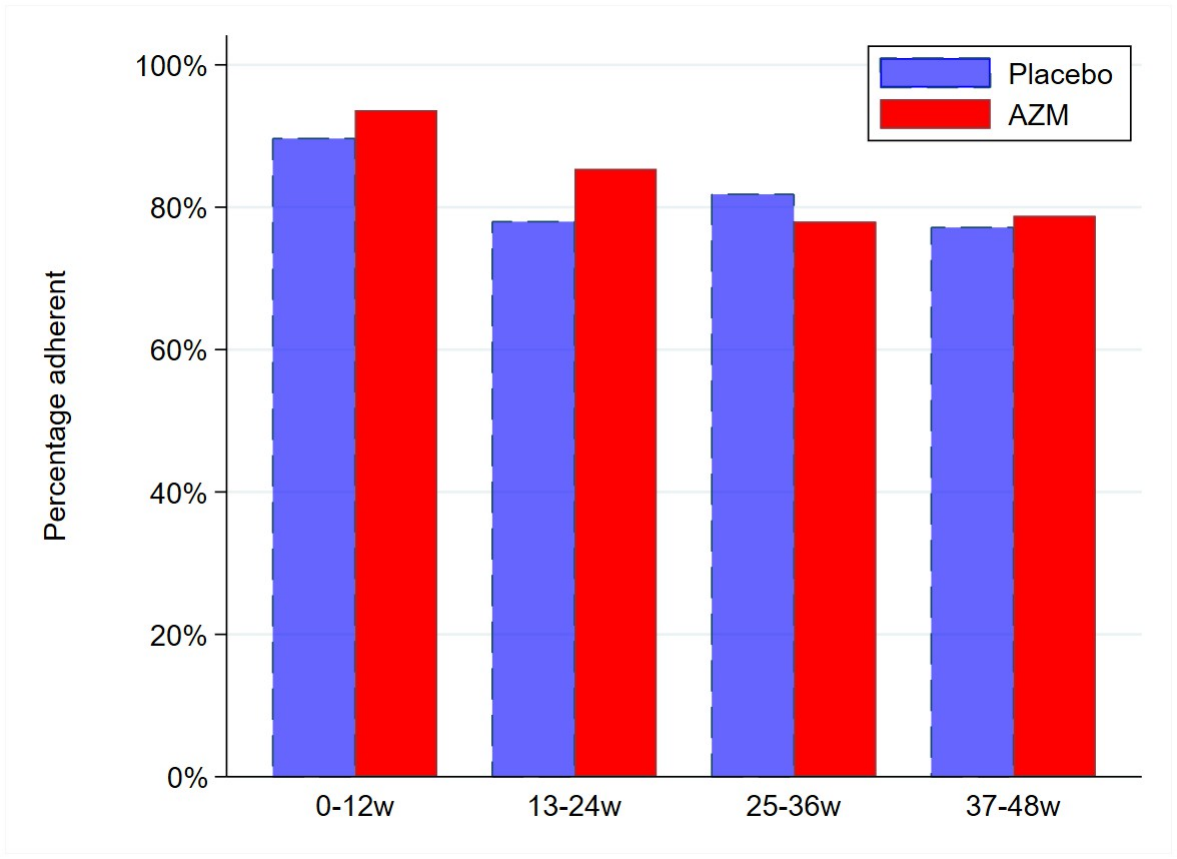
Proportion of participants with good adherence (defined by pill count) over time by trial arm. Score test-for trend—placebo arm p-value = 0.02, AZM arm p-value = 0.0001.

Good adherence, defined as consuming greater than 84.6% (11/13) doses in the time period baseline-12 weeks or 83.3% (10/12) doses in time periods 13–24 weeks, 25–36 weeks and 37–48 weeks, was more prevalent in the azithromycin arm, driven by differences between trial arms up to 24 weeks, decreased over time (Fig 2, Score test for trend p<0.02). Good adherence overall (mean adherence over time periods> 83.7%) was 73.4% in AZM arm, 68.4% in placebo arm and 70.9% overall. Consumption over 115% (more than 15 in first period and more than 14 in other periods) was reported on 85 visits (Table 1).

We included 331 participants with no missing values in our analysis of factors associated with good adherence. Adherent participants were more likely to be Zimbabwean, have supressed HIV viral load, better lung function (FEV_1_ Z-score ≥ -2) and to report ever being treated for TB (Table 2). After adjustment for potential confounding, only the Zimbabwe site (adjusted OR 4.72, 95% CI 2.52, 8.83 compared to the Malawi site) and HIV viral suppression (for those HIV viral load ≥1000 copies/ml adjusted OR 2.08, 95% CI 1.19, 3.63 compared to those <1000 copies/ml) remained associated with better adherence. We did not find any associations between adherence and concurrent pill burden (dose, ART regimen, cotrimoxazole use) or early gastrointestinal side effects (although these were relatively rare).

**Table 2:** Association of baseline characteristics with good adherence.

Characteristics	Level	No. (%) Adherent	Crude OR (95% CI)	p-value	Adjusted OR (95% CI)	p-value
**Trial arm**	Placebo	116 (69.5)	Ref		Ref	
	AZM	124 (75.6)	0.73 (0.45, 1.19)	0.21	0.84 (0.48, 1.45)	0.52
***Socio-demographic***						
**Site**	Zimbabwe	195 (81.3)	Ref		Ref	
	Malawi	45 (49.5)	4.43 (2.62, 7.48)	<0.001	4.72 (2.52, 8.83)	<0.001
**Sex**	Male	129 (75.4)	Ref		Ref	
	Female	111 (69.4)	1.36 (0.84, 2.20)	0.22	1.19 (0.65, 2.14)	0.58
**Age, years**	6–10	35 (74.5)	Ref		Ref	
	11–15	104 (71.2)	1.18 (0.56, 2.49)		0.97 (0.30, 3.07)	
	16–19	101 (73.2)	1.07 (0.50, 2.28)	0.89	1.06 (0.24, 4.66)	0.97
**Attending school currently**	No	43 (72.9)	Ref		Ref	
	Yes	197 (72.4)	1.02 (0.54, 1.93)	0.94	0.76 (0.34, 1.71)	0.51
***HIV and ART***						
**Duration on ART, years**	0.5–< 6	119 (75.3)	Ref		Ref	
	6 +	121 (69.9)	1.31 (0.81, 2.13)	0.28	1.30 (0.34, 1.71)	0.51
**HIV viral suppression, copies/ml**	≤ 1000	149 (79.7)	Ref		Ref	
	> 1000	91 (63.2)	2.28 (1.40, 3.73)	0.001	2.08 (1.19, 3.63)	0.01
**CD4 cell count, cells/mm** ^**3**^	≥ 200	218 (73.4)	Ref		Ref	
	< 200	22 (64.7)	1.51 (0.71, 3.18)	0.29	1.55 (0.65, 3.70)	0.32
***Anthropometry***						
**Dosage (pills/wk)**	1	13 (86.7)	Ref		Ref	
	2	45 (67.2)	3.18 (0.66, 15.33)		2.29 (0.36, 14.59)	
	3	71 (77.2)	1.92 (0.40, 9.21)		1.54 (0.20, 12.17)	
	5	111 (70.7)	2.69 (0.58, 12.41)	0.31	2.16 (0.25, 19.0)	0.61
**Weight-for-age z-score**	Not underweight ≥ -2	111 (69.8)	Ref		Ref	
	Underweight < -2	129 (75.0)	0.77 (0.48, 1.25)	0.29	0.70 (0.36, 1.36)	0.30
**BMI-for-age z-score**	Not thin ≥ -2	194 (72.1)	Ref		Ref	
	Thin < -2	46 (74.2)	0.90 (0.48, 1.69)	0.74	1.14 (0.52, 2.51)	0.75
***Clinical***						
**Ever treated for TB**	No	163 (69.7)	Ref		Ref	
	Yes	77 (79.4)	0.60 (0.34, 1.05)	0.07	0.66 (0.34, 1.29)	0.22
**FEV**_**1**_ **z-score**	≥ -2	141 (76.6)	Ref		Ref	
	< -2	99 (67.4)	1.59 (0.98, 2.58)	0.06	1.59 (0.92, 2.77)	0.10
**Abnormal respiratory rate**	No	134 (71.7)	Ref		Ref	
	Yes	106 (73.6)	0.91 (0.56, 1.48)	0.69	0.83 (0.45, 1.54)	0.56
***Drug use and resistance***						
**ART regimen**	NNRTI-based	174 (71.3)	Ref		Ref	
	PI-based	66 (75.9)	0.79 (0.45, 1.39)	0.42	1.10 (0.56, 2.18)	0.79
**Co-trimoxazole (CTX)**	Yes	216 (72.0)	Ref		Ref	
	No	24 (77.4)	0.75 (0.31, 1.81)	0.52	0.98 (0.38, 2.54)	0.96
**Carriage of bacteria resistant to Azithromycin** ^1^	No	217 (72.8)	Ref		Ref	
	Yes	23 (69.7)	1.16 (0.53, 2.55)	0.70	0.73 (0.29, 1.83)	0.50
***Initial response to study medication***						
**Reported gastrointestinal adverse events** ^2^	No	215 (71.4)	Ref		Ref	
	Yes	25 (83.3)	0.25 (0.19, 1.35)	0.17	0.74 (0.25, 2.22)	0.60

OR < 1 indicate protection from non-adherence

^1^. Carriage in either nasopharyngeal swab or expectorated sputum sample of *Staphylococcus aureus* or *Streptococcus pneumoniae* strains resistant to Azithromycin

^2^. One or more of—nausea, vomiting, diarrhoea or abdominal pain, which are common side effects of treatment with azithromycin—reported as an adverse event in the first 14 days after first dose

**Fig 3:**
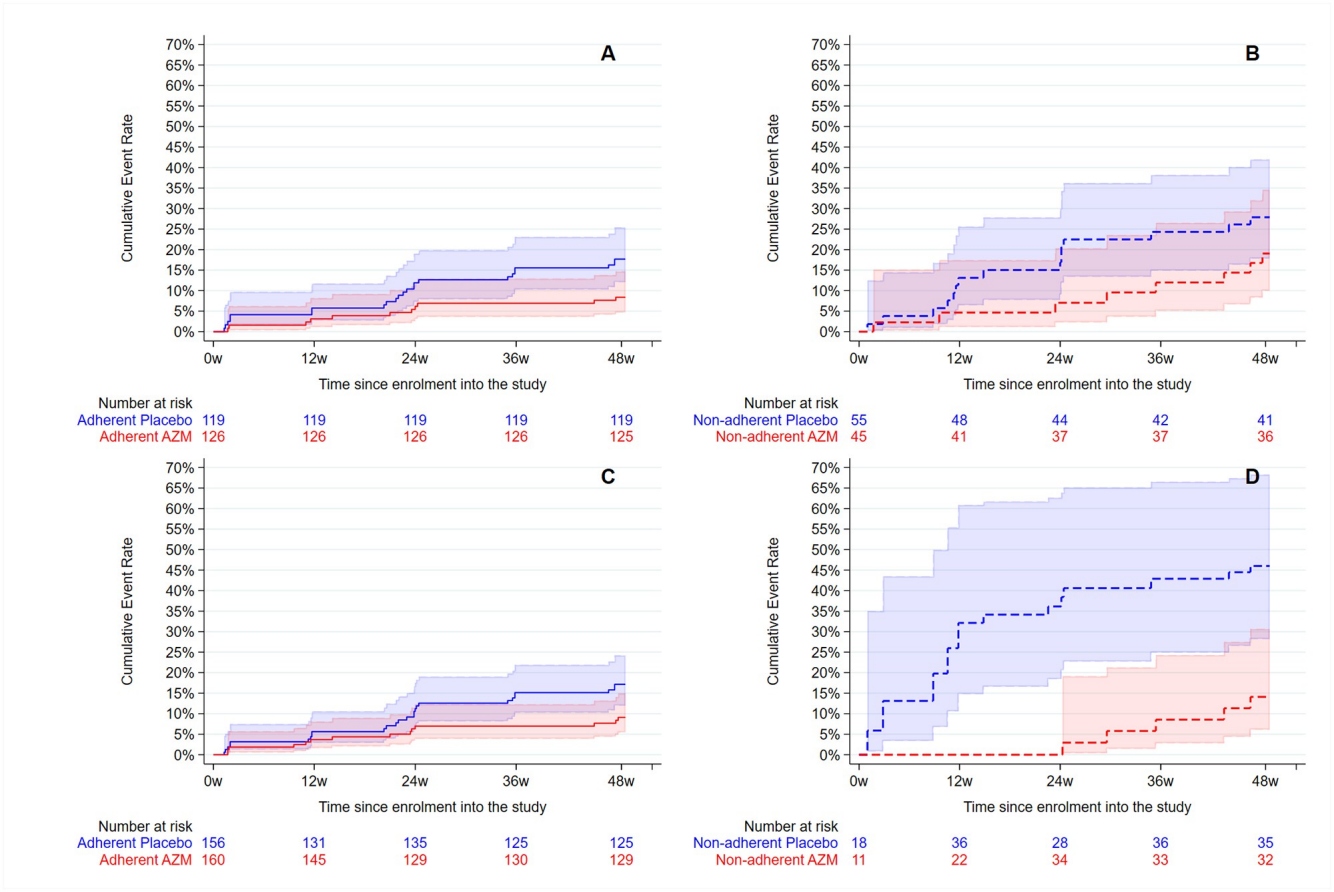
Cumulative incidence and 95% CI of acute respiratory exacerbation by trial arm for (A) adherence averaged over time periods (B) non-adherence averaged over time periods (C) adherence allowed to vary over time and (D) non-adherence allowed to vary over time. Adherent participants are solid lines and non-adherent participants are dashed lines.

Non-adherent participants had a higher rate of ARE than adherent participants (Fig 3). When stratified by adherence, AZM was associated with lower incidence of ARE among adherent participants but there was no evidence for an effect of AZM among non-adherent participants (Table 3), using both an overall measure of adherence (Fig 3A) and a time-varying measure (Fig 3B). However, there was no statistical evidence for heterogeneity in the effect of azithromycin versus placebo by participants who were adherent versus those not adherent (p-values = 0.38 and 0.82).

**Table 3:** Effect of azithromycin on incidence rates for acute respiratory exacerbation and hazard ratios stratified by adherence coverage.

Trial arm	No. episodes/ person years	Rate (95% CI)	Hazard ratio^1^ (95% CI)	p-value
***Adherence coverage over all time periods***				
***Adherent participant*** ^2^				
**Placebo**	23/112	20.6 (13.2, 34.0)	Ref	
**AZM**	11/118	9.3 (5.4, 17.5)	0.42 (0.20, 0.87)	0.02
***Non-adherent participants*** ^2^				
**Placebo**	15/42	35.4 (19.7, 70.4)	Ref	
**AZM**	8/37	21.9 (8.4, 75.7)	0.75 (0.26, 2.16)	0.59
***Adherence coverage varying by time period***				
***Adherent time periods*** ^3^				
**Placebo**	26/128	20.3 (13.4, 32.3)	Ref	
**AZM**	14/132	10.6 (6.5, 18.4)	0.52 (0.26, 1.01)	0.06
***Non-adherent time periods*** ^3^				
**Placebo**	12/26	46.3 (23.0, 109.2)	Ref	
**AZM**	5/23	21.0 (7.9, 85.6)	0.45 (0.15, 1.36)	0.16

^1^. Adjusted for age, sex, site, HIV viral load, season, calendar period and time period in study

^2^. p-value for interaction between trial arm and adherence coverage averaged over time periods = 0.38

^3^. p-value for interaction between trial arm and adherence coverage varying over time periods = 0.82

## Discussion

Among children and adolescents with HIV established on ART, adherence to additional once-weekly medication for management of HIV-associated chronic lung disease was 71% over 48 weeks. A trend was observed that adherence waned over time in study, and was slightly higher among those receiving active treatment (74%) compared to placebo (68.4%). Few studies have investigated the effects of increasing pill burden among children and adolescents living with a chronic condition requiring daily medication, such as for HIV infection, but our findings suggest that it can be feasible for most individuals.

Participants randomised to azithromycin had lower rates of AREs than those on placebo, among adherent and non-adherent participants, although with low statistical power among non-adherent participants; there was no evidence for heterogeneity in the effect of azithromycin versus placebo by adherence. Analysis splitting study time into 12-week periods for which an individual could change their adherence status over time did not materially affect the interpretation of the effect of adherence on ARE. There was limited statistical power to generate efficacy of azithromycin among non-adherent participants, with wide confidence intervals estimated, so a lack of effect of azithromycin compared to placebo could not be ruled out. We observed rates of ARE among participants randomised to the placebo arm who were adherent, which were comparable with those randomised to the azithromycin arm, suggesting that full participation in the trial may have impacted favourably on health outcomes [16]. It is possible that care received by the study team, aside from study medication (such as counselling) contributed to health improvements, and may have attenuated estimates of efficacy related to azithromycin treatment.

Participants whose HIV viral load was not suppressed at enrolment to the trial were less adherent to study medication. Lack of HIV viral suppression is usually a result of poor adherence and poor adherence leads to resistance to ART drugs [17, 18]. It is likely that the challenges faced in adhering to ART will be the same challenges for taking concomitant medications for comorbidities. Additional medication also confers a heavier pill burden which has been found to be a treatment-related barrier to adherence to ART [19]. We did not find evidence that current pill burden (ART regimen, cotrimoxazole use, study medication dose) was associated with adherence.

A past history of TB was associated with better adherence in unadjusted (but not adjusted) models. It may be that these individuals had been used to an additional pill burden (having taken previous anti-TB therapy) and may have found it easier to adhere to additional medication for chronic lung disease. In contrast, having worse lung function (FEV_1_ Z-score <-2) was associated with poorer adherence. The reason for this is not clear. Being unwell was mentioned rarely as a reason for missing study medication.

Reassuringly, presence of gastrointestinal side-effects was not associated with poorer adherence. Rather, overall adherence was higher in the azithromycin arm than in the placebo arm. Little research has been conducted into discontinuation of long-term azithromycin therapy relating to adverse events. In a retrospective assessment of medical records using inverse probability weighting, discontinuation of treatment attributable to gastrointestinal adverse events was <8% for individuals prescribed azithromycin [20]. Compared to other macrolide antibiotics, azithromycin is well tolerated, and minor discomfort does not usually result in discontinuation. It is possible that the presence of side effects reduced the integrity of allocation concealment, resulting in participants guessing their allocation to azithromycin [21]. Uncertainty in model estimates of the effect of GI side effects was high, with relatively large differences in the estimated effects comparing the crude with the adjusted model, which was likely due to their rarity.

There were differences in adherence coverage between sites with Zimbabwe associated with higher adherence coverage compared to Malawi. This could be due to socio-cultural differences, differences in educational level or clinical management practices, or reporting differences. HIV viral suppression was also lower in Malawi, indicating that adherence to ART was also potentially lower than in Zimbabwe.

The major strength of our study was its randomised controlled trial design conducted according to good clinical practice guidelines. Study personal and participants were masked to trial arm, providing care, recording pills consumed, and providing adherence counselling irrespective of the treatment (or placebo) received.

There were practical challenges with dispensing the study medication, which could not be pre-packaged for each individual, as doses were dependent on children’s weight which would change over the course of the study. Weight based prescribing required the research assistant to accurately record the participant’s weight at each visit and ensure the prescriber (in this case, the study physician) had seen the participant’s weight prior to prescribing. In addition, as a cross- check, the weight was also recorded on the drug prescription form for the trial pharmacist to dispense the correct dose per weight. Thirdly, any change in the number of pills since the previous visit i.e. an increase in the number of pills prescribed weekly due to crossing into a new weight band or a decrease due to a loss of weight, was communicated to the participant and the guardian. Over the duration of the trial dispensing errors according to weight and incorrect trial arm were observed, although rarely, and did not impact on the interpretation of the trial findings (in the per protocol analysis) [11]. Drug stockouts were not experienced in the trial and drug expiry was managed by recalling participants.

A limitation of our study was that adherence was based on count of returned pills, and self-report may be subject to desirability bias. Counts were missing for those who withdrew, were lost to follow-up, or forgot to return their tablets when they attended study visits, hence there was potential for misclassification of exposure, although this was rare. We explored two definitions of adherence which were primarily consistent but we did not consider any lagged or cumulative effects of treatment. Our findings cannot be generalised to other conditions or geographical areas. The dosing, pill burden and side-effect profile will vary for other co-morbid conditions. We examined a once-weekly dose which was weight-based with the logistical and practical constraints to administration that brings.

## Conclusion

We found the addition of a weekly weight-based dose of azithromycin for management of co-morbid chronic lung disease was feasible and generally well-adhered to among children and adolescents living with HIV. Management of co-morbidities associated with HIV infections is an emerging priority and will likely become an increasingly important aspect of HIV care. Studies focusing on interventions for addressing comorbidities should learn from existing experience of paediatric HIV management on challenges in adhering to long term medication, and future HIV adherence interventions will need to consider and address the challenges and complexities associated with taking additional medicines over and above lifelong ART.

## Supporting information

S1 Data (XLS)
